# Co‐occurrence patterns in a diverse arboreal ant community are explained more by competition than habitat requirements

**DOI:** 10.1002/ece3.2606

**Published:** 2016-11-23

**Authors:** Flávio Camarota, Scott Powell, Adriano S. Melo, Galen Priest, Robert J. Marquis, Heraldo L. Vasconcelos

**Affiliations:** ^1^ Instituto de BiologiaUniversidade Federal de UberlândiaUberlândiaBrazil; ^2^ Department of Biological SciencesThe George Washington UniversityWashingtonDCUSA; ^3^ Departamento de EcologiaUniversidade Federal de GoiásGoiâniaBrazil; ^4^ Department of Biology and the Whitney R. Harris World Ecology CenterUniversity of Missouri – St. LouisSt. LouisMOUSA

**Keywords:** assembly rules, Brazil, canopy, *Cerrado*, community assembly, niche, species coexistence

## Abstract

A major goal of community ecology is to identify the patterns of species associations and the processes that shape them. Arboreal ants are extremely diverse and abundant, making them an interesting and valuable group for tackling this issue. Numerous studies have used observational data of species co‐occurrence patterns to infer underlying assembly processes, but the complexity of these communities has resulted in few solid conclusions. This study takes advantage of an observational dataset that is unusually well‐structured with respect to habitat attributes (tree species, tree sizes, and vegetation structure), to disentangle different factors influencing community organization. In particular, this study assesses the potential role of interspecific competition and habitat selection on the distribution patterns of an arboreal ant community by incorporating habitat attributes into the co‐occurrence analyses. These findings are then contrasted against species traits, to explore functional explanations for the identified community patterns. We ran a suite of null models, first accounting only for the species incidence in the community and later incorporating habitat attributes in the null models. We performed analyses with all the species in the community and then with only the most common species using both a matrix‐level approach and a pairwise‐level approach. The co‐occurrence patterns did not differ from randomness in the matrix‐level approach accounting for all ant species in the community. However, a segregated pattern was detected for the most common ant species. Moreover, with the pairwise approach, we found a significant number of negative and positive pairs of species associations. Most of the segregated associations appear to be explained by competitive interactions between species, not habitat affiliations. This was supported by comparisons of species traits for significantly associated pairs. These results suggest that competition is the most important influence on the distribution patterns of arboreal ants within the focal community. Habitat attributes, in contrast, showed no significant influence on the matrix‐wide results and affected only a few associations. In addition, the segregated pairs shared more biological characteristic in common than the aggregated and random ones.

## 1 Introduction

A fundamental goal of community ecology is to understand the mechanisms allowing multispecies coexistence in biological communities (Agrawal et al., 2007; Sutherland et al., 2013). To achieve this goal, it is important to assess how species are distributed within communities, and what factors and processes generate these patterns (Chesson, 2000). Niche‐based processes are often offered as an explanation for species distributions within communities, with the general expectation that species must differ in resource use to coexist (Chase & Leibold, 2003; Levine & HilleRisLambers, 2009). Therefore, community assembly may be shaped strongly by biotic interactions among species, and particularly by interspecific competition (Chase & Leibold, 2003; Hutchinson, 1959; MacArthur & Levins, 1967; Schoener, 1974). Nevertheless, the role of interspecific competition in shaping communities is a complex issue that remains under active investigation and debate.

In addressing the role of competition in community assembly, much work has focused on detecting the signature of competition in species co‐occurrence patterns within communities (Gotelli & McCabe, 2002). The central assumption of these analyses is that if competitive interactions scale up to shape community‐level species distribution patterns, nonrandom patterns of localized species co‐occurrence should be observable within a community (Connor & Simberloff, 1979; Diamond, 1975). These analyses are based on statistical null models, which unlike traditional statistical tests, explicitly assess the importance of stochastic processes on community organization (Gotelli & Graves, 1996; Gotelli & Ulrich, 2012). Moreover, a null model approach allows the inclusion of constraints to preserve features of the empirical data, which cannot be done easily in parametrized statistical tests (Gotelli & Ulrich, 2012). These kind of analyses are particularly valuable because they facilitate the use of large biodiversity‐survey datasets to address the underlying processes of community assembly (Gotelli, 2000; Gotelli & Graves, 1996). However, it is important to note that nonrandom patterns of species co‐occurrence are not necessarily the result of competitive interactions (Connor & Simberloff, 1979, 1984; Peres‐Neto, Olden, & Jackson, 2001). Other factors not directly related to competition, such as dispersal ability and habitat requirements, can produce similar patterns (Azeria, Ibarzabal, & Hébert, 2012; Sanderson, 2004; Sfenthourakis, Tzanatos, & Giokas, 2006). It is also possible that interspecific niche similarities can be more important in shaping community assembly than niche differences, a process known as “niche filtering” (Fowler, Lessard, & Sanders, 2014).

Given the various potential influences on community assembly, co‐occurrence analyses become more valuable if they can differentiate between the signature of competition and other factors (Connor & Simberloff, 1979; Sfenthourakis et al., 2006). Once again, a null model approach is ideal to tackle this issue, as its flexibility allows the explicit incorporation of the variables of interest in the analysis (Gotelli & Graves, 1996). Nevertheless, co‐occurrence analyses are typically applied to large datasets that lack the sampling structure to address other influences on co‐occurrence patterns (Gotelli, 2001). Additionally, co‐occurrence patterns are usually assessed only at the whole‐community level, which ignores potentially important interspecific interactions (Diamond & Gilpin, 1982) and weakens inferences about the factors that are responsible for the observed patterns (Sanderson, 2004; Veech, 2014). This is because we need to know the negatively or positively associated species pairs to compare the biological traits and habitat affinities that may explain the observed association patterns (Veech, 2014). To learn more from species co‐occurrence data, it is therefore necessary to use null model analyses that incorporate potentially important environmental influences, and contrast whole‐community versus pairwise patterns (Gotelli, 2001; Ulrich & Gotelli, 2013).

Ants are abundant, highly diverse, and ecologically important in tropical systems. Moreover, ants engage in obvious and often intense competitive interactions (Hölldobler & Wilson, 1990; Lach, Parr, & Abbot, 2010). These attributes, combined with the relative ease with which communities can be sampled, make them especially well suited for use in co‐occurrence analyses. Not surprisingly, many studies have evaluated co‐occurrence patterns in ant communities (Gotelli & McCabe, 2002), particularly in tropical arboreal ant communities (Dejean et al., 2010; Pfeiffer, Cheng Tuck, & Chong Lay, 2008; Sanders, Crutsinger, Dunn, Majer, & Delabie, 2007). Indeed, arboreal ant communities have been seen as a classic example of a nonrandom pattern of species co‐occurrence (Blüthgen & Stork, 2007). More specifically, it has been hypothesized that these communities have a “mosaic distribution,” where two or more dominant ant species maintain mutually exclusive territories, and each coexists with a specific set of subordinate and subdominant ant species (Jackson, 1984; Leston, 1970). This mosaic distribution pattern is proposed as a repeatable, specific outcome of intense competition between dominant species over foraging territories (Blüthgen & Feldhaar, 2010; Blüthgen & Stork, 2007). Nevertheless, the use of co‐occurrence analyses to address the role of competition in structuring arboreal ant communities, and especially the specific case of the “ant mosaic hypothesis,” has been criticized heavily. This criticism was based on the shortcomings of analytical methodologies for considering other influences on nonrandom co‐occurrence patterns (Gotelli, 2001; Ribas & Schoereder, 2002).

To avoid past issues with co‐occurrence analyses of arboreal ant communities, it is necessary to analyze a suite of factors that may produce nonrandom co‐occurrence patterns, but that are not directly related to competition. Environmental factors are a particularly important focus in this regard, because they are often reflected in the habitat use of different species. For arboreal ants, a single tree is a distinct habitat patch, and tree size, tree species, and the surrounding vegetation can be considered key habitat attributes (Pacheco & Vasconcelos, 2012; Ribas, Schoereder, Pic, & Soares, 2003). In fact, trees of different sizes typically host different abundance and richness of arboreal ant species (Campos, Vasconcelos, Ribeiro, Neves, & Soares, 2006; Koch, Camarota, & Vasconcelos, 2016; Powell, Costa, Lopes, & Vasconcelos, 2011). Similarly, some ant species may nest only on specific tree species (Klimes et al., 2012; Sanders, Gotelli et al., 2007), and the structure of the surrounding vegetation can also have a significant impact of species richness (Powell et al., 2011). With all these aspects considered, it is possible that a nonrandom pattern of species associations may result from distinct habitat use and not interspecific competition, as it is often claimed. Nevertheless, most arboreal ant datasets are not structured in a way that allows for co‐occurrence analyses to systematically address the potential influence of environmental factors on community assembly.

In this study, we assess the extent to which species coexistence patterns in arboreal ants can be explained by interspecific competition or by habitat associations. We further address whether the biological characteristics of the most common ant species, such as nesting preferences and recruitment strategies, can explain the patterns we uncover. We achieved this with data from a Neotropical savanna, where the natural habitat allowed for an extensive biodiversity survey structured by tree size, tree species, and vegetation structure, and where the biological characteristics of ant species were quantifiable. To analyze these data, we used a suite of null models that first account only for the species incidence in the community, and then explicitly incorporate habitat attributes. We further performed all analyses first with all species in the community, and then with only the most common species. Moreover, as the community‐wide analyses can hide relevant species interactions (Veech, 2014), we also used pairwise analysis to search for co‐occurrence patterns of different species pairs. Following this, we assessed similarities in key biological properties of the most common ant species in order to interpret the observed pairwise patterns. We used these analyses to address three main questions: (1) do co‐occurrence patterns in our focal arboreal ant community deviate significantly from that expected under a process of random community assembly? (2) to what extent do these co‐occurrence patterns change when we account for potentially important habitat attributes? (3) to what extent can the observed co‐occurrence patterns be explained by differences or similarities in key traits of the species involved?

## 2 Materials and Methods

### 2.1 Study area

This study was conducted at the Reserva Ecológica do Panga, a 404‐ha reserve located 35 km south of Uberlândia, Minas Gerais, Brazil (19°10′ S, 48°23′ W). The reserve is located in the Neotropical savannas of central Brazil (*Cerrado* hereafter). Cerrado is characterized by a vegetation mosaic dominated by savannas of variable structure (Oliveira‐Filho & Ratter, 2002). This study was conducted in two kinds of savannas with marked differences in their vegetation structure: the *cerrado ralo*, with scattered distributed trees and higher grass cover, and the *cerrado sensu stricto*, with a higher tree density and lower grass cover.

### 2.2 Ant sampling

We collected ants on a total of 240 trees, with even sampling of 40 trees for each of the following six tree species: *Caryocar brasiliense* Cambess. (Caryocaraceae), *Qualea grandiflora* Mart. (Vochysiaceae), *Stryphnodendron polyphyllum* Mart. **(**Fabaceae**)**,* Sclerolobium aureum* (Tul.) Benth. (Caesalpiniaceae), *Machaerium opacum* Vogel (Fabaceae), and *Kielmeyeria coriacea* Mart. & Zucc. (Clusiaceae). These species are common in the study area, and in the Cerrado as a whole (Moreno, 2005). Half of the trees were sampled in areas of *cerrado ralo* (open savannas hereafter) and the other half in areas of *cerrado sensu stricto* (closed savannas hereafter). Each tree species had the same number of individuals sampled in each vegetation type. Trees from each species were classified as “small,” “medium,” or “large” based on trunk diameter 10 cm above the soil surface. Small trees had a diameter up to 11.9 cm (60 trees), medium trees were between 12 and 21.9 cm in diameter (121 trees), and large trees had a diameter of 22–36 cm (59 trees).

We used baited arboreal pitfall traps for the ant sampling, following the procedure of Powell et al. (2011). Briefly, the number of traps per tree ranged from 4 to 10, according to the size of the tree. On each tree, half of the traps contained honey diluted in water (1:7) and the other half contained human urine (diluted 1:2 in water). A small quantity of detergent was included in the bait liquids to break the surface tension and thus improve the killing efficiency. Each pitfall trap consisted of approximately 20 ml of bait liquid in an 80‐ml plastic cup (6 cm height and 5 cm diameter). The traps were distributed throughout the crown of each focal tree, using wire to hold the rim horizontal and touching a branch. Traps were left on the trees for 48 h for the collection of both diurnal and nocturnal ant species. All sampling was completed over a period of 10 days, using a stratified sampling procedure of four trees of different sizes per species on each day, for a total of 24 trees sampled each day. The contents of all traps were sorted and identified to species and morphospecies in the laboratory. All material was compared against the existing ant collection at the Federal University of Uberlândia, which is built from extensive collections at many Cerrado sites. Voucher specimens of all species/morphospecies are held at the Zoological Collection of the Federal University of Uberlândia in Brazil.

### 2.3 Null model analyses

#### 2.3.1 Co‐occurrence metrics

To assess species co‐occurrence pattern, we used a standardized version of the C‐score of Stone and Robert (1990) (St. C‐score), which corrects for the differences in species incidence within the community (Azeria et al., 2009). The C‐score measures the mean number of checkerboard units between all possible pairs of species in a data matrix (Stone & Robert, 1990). It can, however, also be used for analysis between a single pair of species. When compared to other indices used to assess co‐occurrence, the C‐score has a smaller probability of type I and II errors (Gotelli, 2000). The number of checkerboard units (CU) for each species pair in the standardized version of the index is: *SCU = *(*r*_*i*_ *− A*)(*r*_*j*_ *− A*)/(*r*_i_*r_*j*_), where *A* is the number of shared sites (trees) and *r*_*i*_ and *r*_*j*_ are the number of trees on which each species *i* and *j*, respectively, are found (Stone & Robert, 1990). We also used the Sørensen index (SOR) (Dice, 1945) to measure the mean number of shared sites (i.e., trees in our study) between the different pairs of species: The index is calculated as *SOR = *2*A*/(2*A + B + C*) where *A* represents the number of shared sites between species *r* and *j*, while *B* represents the number of sites where species *r* is present and species *j* is absent, and *C* is the converse of *B*.

The magnitude of the standardized C‐score and the Sørensen index depend on the frequency of occurrence of the species, precluding a direct interpretation of the observed values. Accordingly, observed values of these statistics can be assessed by comparing them to a distribution generated by a null model. This is done by calculating a standardized effect size (SES), so that results are comparable across communities, pairs of species, and even with other studies. The SES is calculated as follows: SES = (observed index value *−* mean of simulated index values)/standard deviation of simulated index values. The significance of the observed SES values was computed as the proportion of simulated values equal to or more extreme than the observed value (Gotelli, 2000). SES values greater than 1.98 (*p* < .05) indicate a segregated distribution under the St. C‐Score and aggregated distribution under the SOR and vice‐versa when SES values are <−1.98.

We assessed species co‐occurrences using the fixed–fixed (FF) null model, where the frequencies of occurrence (fixed columns) and the original species richness on each tree are maintained (fixed rows). Thus, differences among sites are maintained, but the species occurrences are random with respect to one another, which makes it appropriate for detecting patterns of species interactions (Gotelli, 2000). Furthermore, the FF null model is not vulnerable to type I error, has power in the face of noisy data, and measures a pattern of co‐occurrence consistent with competitive exclusion (Gotelli, 2000).

#### 2.3.2 Unconstrained versus habitat‐constrained null models

We further implemented two different classes of the FF null model: one that takes into account only aspects of the species occurrence and site species richness of the original matrix (“unconstrained null models”), and one that incorporates the species specificity for a given habitat on the species null distribution (“habitat‐constrained null models”) (Azeria et al., 2012; Sfenthourakis et al., 2006). The unconstrained model was contrasted against three different habitat‐constrained models, each using a different environmental variable. The environmental variables used in the habitat‐constrained models were as follows: tree species (*Caryocar brasiliense*,* Qualea grandiflora*,* Stryphnodendron polyphyllum*,* Sclerolobium aureum*,* Machaerium opacum*,* Kielmeyeria coriacea*), tree size (small, medium, large), and vegetation structure (open or closed). These three different environmental variables are related to the host tree characteristics and were chosen for their potential impact on ant species richness and composition (Djiéto‐Lordon, Dejean, Gibernau, Hossaert‐McKey, & McKey, 2004; Klimes et al., 2012; Powell et al., 2011).

The constrained null models differ from their unconstrained counterparts by restricting the randomization of occurrence values to the same level of the constraining factor. For instance, an ant species record found on the level “*Caryocar brasiliense”* of the factor tree species will be randomized among individuals of “*Caryocar brasiliense.”* It will not be assigned, during randomizations to generate simulated communities, to a different host plant species.

In the case where one of the two indexes (e.g., St. C‐score) is significantly different from the null distribution under both the unconstrained and habitat‐constrained models, this would suggest that some interspecific interaction is indeed helping to explain the observed pattern. On the other hand, if the index is significant under the unconstrained model but not under the habitat‐constrained model, it suggests that the habitat selection is more important for explaining the observed patterns than competition (Azeria et al., 2012; Peres‐Neto et al., 2001). Another possible outcome is that species pairs that are not significant under unconstrained analyses can be significant under habitat‐constrained null models. This may indicate that there is a negative association *within* a shared habitat (for segregated pairs) or that the differences in species affinity for a given habitat could have masked an otherwise positive interaction (for aggregated pairs) (Azeria et al., 2012).

#### 2.3.3 Matrix‐level versus pairwise‐level approaches

Most of the methods used for co‐occurrence analyses can be classified as either “matrix” or “pairwise” approaches. The matrix approach calculates the co‐occurrence metrics as a property of the whole presence/absence species matrix (Gotelli, 2000; Pitta, Giokas, & Sfenthourakis, 2012). We first calculated null models for whole matrices and then performed pairwise analyses to assess the co‐occurrence for each species pair separately. This was done to determine whether each possible pair in the community has an aggregated, segregated, or random co‐occurrence pattern (Gotelli & Ulrich, 2012; Pitta et al., 2012; Veech, 2014). We present data for the two indices considered (St. C‐score and Sorensen) on the matrix‐level analyses. However, for the pairwise analyses we present results for only the St. C‐score, as the outcomes were more numerous and qualitatively identical across both indices.

For the whole matrix approach, we conducted analyses first including all sampled ant species (75 spp.) and then only the 14 most common ant species. For the pairwise approach, we conducted analyses only with the 14 most common ant species. Restricting the pairwise analyses to the common species avoids the loss of statistical power associated with including species that were rare. These 14 ant species were chosen because they were found in at least 20 of the sampled trees. These 14 ant species also included the most abundant species in the community, representing 93% of the total number of species incidences across all sampled trees.

#### 2.3.4 Matrix randomizations

We ran 5,000 randomizations for each null model. Randomizations were done using the function “commsimulator” in R environment version 3.3.3 (R Development Core Team 2014), available in the “vegan” package (Oksanen, Kindt, Legendre, O'Hara, & Stevens, 2013). The simulated communities were obtained using the “quasiswap” algorithm, where each simulation uses the original matrix and not the previous randomized matrices (Gotelli & Entsminger, 2001, 2003). We wrote R routines for the pairwise analyses, and the habitat‐constrained analyses.

### 2.4 Species–habitat associations

We assessed whether ant species were associated with a particular tree species, tree size, or trees in the open or closed areas using Indicator Species Analysis (Dufrene & Legendre, 1997). This analysis estimates the strength of the association of different species with distinct groups of sites (Cáceres & Legendre, 2009). We used the IndVal program of Dufrene and Legendre (1997) and assessed the significance of the indicator values for each species using Monte Carlo′s permutation tests with 5,000 randomizations.

### 2.5 Nesting and foraging ecology of the most common ant species

#### 2.5.1 Ant nesting ecology

To assess the nesting ecology of the 14 most common species, we opened and measured stems of different sizes belonging to 20 trees of each of the six tree species studied. In each tree, we removed six branches of a given base diameter size and then dissected each base stem and all subsequent stems up to the terminal tips. We set a base diameter of approximately 2 cm for eight trees and a base diameter of 5 cm for 12 of the sampled trees. For each ant nest found, we measured the mean length of the nesting cavity. Nests were further classified as being in dead or live stems, with cavities that were small, medium, large, or very large in length, or simply under the tree bark. Small cavities were up to 5 cm of length, medium cavities from 5.1 to 25 cm, large cavities those from 25.1 to 50 cm, and very large cavities those with more than 51 cm of length. As the percentage of the tree occupied by an ant species can be important to define its dominance status, we also calculated the occupation rates of the available cavities by the different ant species. For this, we summed the total length of used cavities by each species and divided by the total length of available cavities on the trees where each species was found. Those species that occupied more than 25% of the available cavities were considered as having “extensive cavity use.”

#### 2.5.2 Ant activity schedule and recruitment to baits

We observed ant activity at baits on 175 trees during the day (between 08:00 and 12:00) and a subset of 44 of these trees during the night (between 19:00 and 23:00). These were different trees from those used in the ant survey (above), and the baits were solid pieces of sardine. Observations were made on 8–10 trees each day until observations had been made on all trees. For each tree, activity at the baits was observed within ten minutes of the baits being placed on the tree, and then again after one, two, and three hours. Ant species were classified as “diurnal” if they were only or mostly seen foraging during the day, “nocturnal,” if they were seen only or mostly during the night and “both” if they were seen foraging both day and night. We also recorded the maximum number of ants of each species present on a given bait across the four observations periods. Recruitment strength was then summarized into four different categories for the analyses: “small,” between one and five workers, “medium,” from 6 to 10 workers, “large,” from 11 to 25 workers, and “very large,” with more than 25 workers.

#### 2.5.3 Differences between segregated and aggregated pairs

To assess differences in dissimilarity in traits between species that formed segregated and aggregated pairs, we performed a logistic regression, with the dissimilarity (Sørensen) between the species pairs as the predictor variable and the kind of association (1 = segregated, 0 = aggregated) as the response variable. For this analysis, we excluded those species pairs that were determined by habitat variables (one segregated and two aggregated pairs).

## 3 Results

### 3.1 Description of the community and ecology of the most common species

Overall, we collected 75 ant species from 17 genera (Table S1). Most ant species were very low in frequency in the community, with only 14 species occurring on at least 20 of the 240 sampled trees. The most frequent species included five species of *Camponotus*, four species of *Pseudomyrmex*,* Azteca* sp. 1, *Cephalotes pusillus*,* Crematogaster ampla*,* Solenopsis* sp. 1, and *Tapinoma* sp. 1. The nesting and foraging ecology of these species are presented in Table 1. Most species of *Pseudomyrmex* were strictly diurnal, recruited very few workers to baits, and nested exclusively in small branches. Among the *Camponotus* species, *Ca. melanoticus* was only found nesting in cavities located in live branches of medium size, *Ca. sericeiventris* was only in cavities located in large and very large sized live branches, whereas the remaining three species nested in branches of small, medium, and large size (Table 1). Three of the *Camponotus* species were strictly nocturnal, whereas the remaining two forage during the day and the night. Five of the 14 species had extensive cavity use, but only three of those (*Azteca* sp. 1, *Crematogaster ampla*, and *Cephalotes pusillus*) had large colonies whose nests are located in live and dead branches of any size.

**Table 1:** Ecological characteristics of the 14 most common ant species in the study area

Ant species	Species ecological characteristics											
	Nesting ecology										Other	
	Live small branches	Dead small branches	Live medium branches	Dead medium branches	Live large branches	Dead large branches	Live very large branches	Dead very large branches	Under bark	Extensive cavity use	Activity period	Recruit.
*Azteca* sp. 1	x	x	x	x	x	x	x	x		Yes	Both	XL
*Ca. melanoticus*					x					No	Nocturnal	M
*Ca. sericeiventris*					x	x	x	x		Yes	Both	M
*Ca. atriceps*			x	x	x	x	x	x		No	Nocturnal	L
*Ca. bonariensis*			x	x	x	x				No	Nocturnal	L
*Ca. senex*	x	x	x	x	x	x				No	Diurnal	M
*Ce. pusillus*	x	x	x	x	x	x	x	x		Yes	Both	L
*Cr. ampla*	x	x	x	x	x	x	x	x		Yes	Both	XL
*P. curacaensis*	x	x	x	x						No	Diurnal	S
*P. elongatus*	x	x	x	x						No	Both	S
*P. gracilis*	x	x	x	x						No	Diurnal	S
*P. urbanus*	x	x	x	x						No	Diurnal	S
*Solenopsis* sp. 1	x	x	x	x					x	Yes	Both	XL
*Tapinoma* sp. 1	x	x	x	x					x	No	Both	XL

On the Recruitment size, S = small, M = medium, L = large, and XL = very large recruitment rates.

With the exception of *Azteca* sp. 1, which was found mostly on *S. aureum* trees (Table 2), none of the remaining species had a significant association with a given tree species (Indicator Species Analysis, Table 2). Similarly, with the exception of *Ca. sericeiventris*, which was mostly found on trees located in the closed savanna habitats, none of the remaining ant species showed a significant association with a given vegetation structure. However, four of the 14 species showed significant associations with trees of a given size (Table 2).

**Table 2:** Species–habitat association, with the IndVal results of the 14 most common arboreal ants on our study area

Ant species	Tree species	Tree size	Vegetation structure
*Camponotus senex*			
*Pseudomyrmex gracilis*			
*Cephalotes pusillus*		26.24* (small)	
*Camponotus bonariensis*		26.27* (small)	
*Camponotus atriceps*		21.86** (large)	
*Tapinoma* sp. 1			
*Azteca* sp. 1	13.14* (SA)		
*Camponotus melanoticus*			
*Pseudomyrmex curacaensis*			
*Camponotus sericeiventris*			12.97* (closed)
*Pseudomyrmex elongatus*			
*Solenopsis* sp. 1			
*Crematogasters ampla*			
*Pseudomyrmex urbanus*		11.83*** (small)	

Between brackets are the habitat characteristics most associated with a given ant species (numbers with *indicates values of *p* < .05, with **p < .01 and with ***p < .005). Empty spaces mean that there was not a significant relationship between a given ant species and a habitat characteristic.

### 3.2 Null model analyses

#### 3.2.1 Matrix‐level co‐occurrence analyses

The analyses with all 75 ant species in the community showed random patterns under the unconstrained models for the two indexes used here (Table 3). Similarly, the habitat‐constrained models showed random patterns of co‐occurrence (Table 3). When we considered only the 14 most common ant species in the community, the two indexes showed a significantly segregated pattern of species associations (Table 3).

**Table 3:** Co‐occurrence patterns at the community level of arboreal ant under unconstrained and habitat‐constrained analyses

Species matrix	Unconstrained	Habitat‐constrained		
		Tree species	Tree size	Vegetation structure
(a) All 75 ant species				
SES of St. C‐score	−0.93	−1.11	−1.06	−1.03
SES of Sorensen	0.61	0.32	0.34	0.79
(b) The 14 most frequent ant species				
SES of St. C‐score	**4.27** *	**4.21** *	**4.28** *	**4.2** *
SES of Sorensen	−**3.87** *	−**3.65** *	−**3.75** *	−**3.67** *

Negative values of standardized effect size (SES) indicate aggregation between species pairs under the St. C‐score and segregation under the Sorensen index. Positive values of the St. C‐score and negative values of the Sorensen index indicate segregation between species pairs (SES in bold and with * indicates values of *p* < .05).

#### 3.2.2 Pairwise‐level co‐occurrence analyses

**Figure 1:**
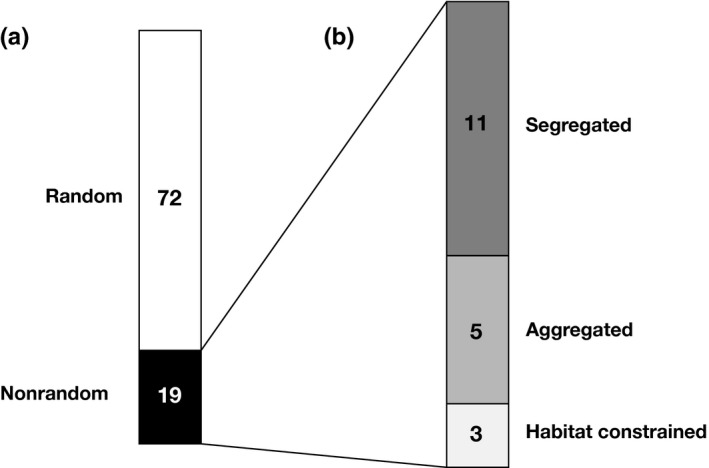
Number of ant species associations in pairwise analyses of the 14 most frequent species in the focal community. (a) Random and nonrandom associations and (b) within the nonrandom associations the segregated, aggregated, and habitat‐constrained associations

Our pairwise analyses of the 14 most frequent species showed that 21% of the pairs analyzed (19 of 91) have significantly nonrandom co‐occurrence patterns under either the constrained or unconstrained null models (Figure 1a). Of these nonrandom pairwise species associations from the unconstrained model analyses, 11 were segregated and five were aggregated, while three become nonsignificant under one or more constrained models, indicative of the associations being habitat‐constrained (Figure 1b). Finally, only one of the species pairs that were not significant under the unconstrained models became significant with the habitat‐constrained models (Table S2).

#### 3.2.3 Differences between segregated and aggregated pairs

**Figure 2:**
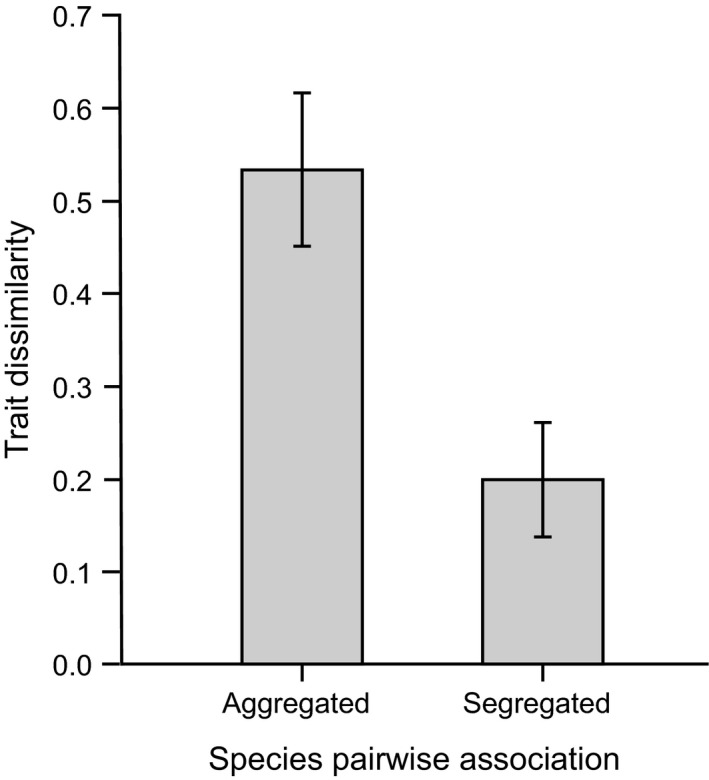
Mean trait dissimilarity (Sørensen ± SE) between aggregated and segregated species pairwise associations

We found a significant difference in the mean distance in trait dissimilarities between the segregated and aggregated pairs (*t* = 1.97, *p* < .05, *N* = 16). The segregated pairs had a lower mean dissimilarity than the segregated ones (Figure 2).

Of the ten pairs that were segregated under both the unconstrained and constrained models, both species of the pair tended to have similar nesting/foraging ecology (Table S3). For instance, both *Azteca* sp. 1 and *Crematogaster ampla* forage during the day and the night, nest in live and dead branches of any size, recruit massively to baits, and have extensive cavity use and large colonies (Tables 1 and S3). Similarly, *Pseudomyrmex gracilis* and *Pseudomyrmex urbanus*, which also had a significantly segregated co‐occurrence pattern, are strictly diurnal species that only nest in small and medium branches. In contrast, species that formed aggregated pairs rarely shared the same nesting or foraging characteristics (Table S3).

## 4 Discussion

We assessed the potential role of interspecific competition and habitat selection on the organization of an arboreal ant community in a Neotropical savanna. We did this by taking advantage of a dataset that took into account the characteristics of the host trees and abundance of ant species in the community. The matrix‐level co‐occurrence analyses showed random pattern of species associations when all ant species were considered, but a significant segregated pattern when using only the 14 most common ant species. Moreover, the pairwise analyses indicated a significant number of segregated and aggregated species associations among these most common ant species. Few of the significant segregated associations became nonsignificant when we took into account the species, size, or the connectivity of the host tree. Concordantly, few species showed significant associations with a given tree species, or with trees of a given size or vegetation structure. Overall, our results suggest that these segregated associations are more consistent with competitive interactions between species driving species co‐occurrence patterns, with a limited influence of habitat selection. This result was further supported by our comparisons of the nesting and foraging ecology of the species that formed segregated pairs. Species that formed these pairs almost always shared the same nesting ecology, foraging ecology, or both, whereas those that formed aggregated pairs did not.

### 4.1 Matrix‐ versus pairwise‐level analyses

The matrix‐level co‐occurrence analyses failed to detect any pattern of species association when we accounted for all 75 ant species in the community. These results are concordant with other studies assessing local ant co‐occurrence patterns at the community level in natural habitats, especially recent ones using more robust null models (e.g., Campbell, Fellowes, & Cook, 2015; Gotelli & Ellison, 2002; Sanders, Gotelli et al., 2007; Stuble et al., 2013). However, matrix‐level analyses represent an average of values calculated for all individual pairs of species (Gotelli, 2000). Therefore, important species interactions in the studied community may be obscured in analyses with many species (Gotelli & Ulrich, 2012), as strong interactions can be diluted by weaker ones and even cancel each other out if both positive and negative interactions exist (Diamond & Gilpin, 1982; Sanderson, 2004). This may help to explain why contrasting results were obtained in the analyses involving the whole community and those using only the 14 most common ant species. In the latter case, strong evidence of a segregated co‐occurrence pattern was obtained.

Furthermore, with the pairwise analyses, we found a relatively large number (~21%) of nonrandom species associations, either segregated or aggregated, thus well above the number expected to occur by chance alone (5%). In an analysis of species‐pairs associations from 30 published matrices, Sfenthourakis et al. (2006) showed that only eight matrices had a number of deviations (segregation or aggregations) higher than 5%, with just three matrices having more than 10%. Our results and those of Sfenthourakis et al. (2006) clearly indicate the need of both matrix‐ and pairwise‐level analyses to the assessment of species associations in a community. Moreover, it also suggests the possibility of novel results with the use of a pairwise approach in a reassessment of already published arboreal ant co‐occurrence matrices.

### 4.2 Habitat attributes

The detection of a significant species association has been frequently used as sufficient evidence to infer a specific ecological process, and especially competition. Nevertheless, different processes may produce the same pattern, leaving these inferences open to criticism (Gotelli & Graves, 1996; Strong, Simberloff, Abele, & Thistle, 1984). The structured nature of our dataset allowed us to take the next step and evaluate the relative importance of host tree selection in explaining the observed patterns. Here, we have found that when information about the host trees was taken into account, the results of our analyses changed very little. In fact, the significance of the observed matrix‐wide analyses did not change with the inclusion of host tree attributes for the 14 most common ant species. Moreover, only a small proportion (15.8%) of the significant pairwise species associations became nonsignificant with the inclusion of host tree characteristics. This indicates that, for arboreal ants, species aggregations are rarely due to shared host tree preferences. Similarly, in most cases, species segregations could not be attributed to distinct host tree preferences between ant species. These results probably reflect the fact that none of the most common species in our community showed strong affinities for trees from a given vegetation structure, size or species (Table 2). Our results contrast with those of Azeria et al. (2012) who showed that co‐occurrence patterns of saproxylic beetles are largely modulated by the species and size of the host tree, rather than by interspecific competition.

### 4.3 Species traits

Broadly, our results indicated a number of significant positive and negative pairwise species associations, but with no community‐wide signature and no real influence of host tree attributes. The significant associations are therefore better explained by competition among specific pairs of species, and we may need to turn to the traits of the ants as an explanation. Indeed, our data give support to the prediction that trait‐mediated competitive processes are important in explaining interspecific associations (Adler et al., 2014; Chesson, 2000), as the co‐occurring species (aggregated) had fewer similarities in common than the segregated species.

Nesting sites can be a limited resource for cavity‐nesting arboreal ants (Blüthgen & Feldhaar, 2010; Philpott & Foster, 2005; Powell et al., 2011), with many ant species showing some level of specialization over the use of these resources (Powell et al., 2011). Our data showed that more than half of the segregated pairs of species have marked similarities in their nesting habits, and this similarity can be related either to the structure and/or the extensive use of their nests. In contrast, all coexisting species pairs had markedly different nesting habits. Campbell, Fellowes, and Cook (2013) also showed the importance of nest site selection on domatia‐dwelling ant species coexistence.

Another important aspect in defining the directions of interspecific associations was the activity period of the ant species. Almost half of the negatively associated pairs were formed by species foraging on the same time schedule. Indeed, there are a number of studies showing the importance of activity time for ant species coexistence (Cerdá, Retana, & Cros, 1997; Cerdá, Retana, & Manzaneda, 1998; Lessard, Dunn, & Sanders, 2009; Stringer, Haywood, & Lester, 2007). In fact, subordinate species have been shown to be better adapted than the dominant ones for foraging at extreme temperatures, avoiding direct interactions with the dominant species and the possible exclusion from a habitat patch (Bestelmeyer, 2000). This pattern has been rarely tested in tropical habitats (Wittman et al., 2010), but our results suggest that it may also be important in tropical arboreal ant communities.

Ant recruitment rates also appear to be important in explaining the directions of interspecific associations. More specifically, the recruitment rates were almost always different between coexisting (i.e., aggregated) species, which can mean that there is a trade‐off between these ants on the ability to find new resource and to dominate or even monopolize them. In this trade‐off, also known as “discovery dominance,” ants usually differ on their recruitment ability, with superior competitors having lower recruitment rates and inferior ones investing all the energy of the colony in finding resources (Fellers, 1987; Parr & Gibb, 2012). The extent to which these kinds of trade‐offs are important in tropical arboreal ant communities can therefore be seen as a valuable focus for future work.

Despite focusing on different aspects of the foraging habits of different ant species, it is important to note that we did not take into account dietary preferences of the different ant species as an important trait to explain ant species coexistence. However, most arboreal ants have diets based on sugar‐rich exudates (i.e., honeydew and extrafloral nectar), acting as functional herbivores, with very few cases of true predators (Blüthgen, Gebauer, & Fiedler, 2003; Davidson, Cook, Snelling, & Chua, 2003; Russell et al., 2009). Moreover, the presence of food seems to not be a major axis of niche differentiation, with only limited effects on the structure of arboreal ant communities in the focal habitat of this study (Camarota, Powell, Vasconcelos, Priest, & Marquis, 2015).

### 4.4 Competition in arboreal ant communities versus the ant mosaic hypothesis

Ant communities are often thought to be structured by interspecific competition (Hölldobler & Wilson, 1990; Parr & Gibb, 2010), but many assumptions allowing for such interpretation still need to be properly tested (Cerdá, Arnan, & Retana, 2013). One of these assumptions states that arboreal ant communities are spatially distributed in a mosaic formed by the mutually exclusive foraging territories of dominant ant species (Leston, 1970; Majer, 1972; Room, 1971). In the classical examples of ant mosaic, the competition over territory is limited to behaviorally dominant species with large colonies (Bluthgen & Stork, 2007). In our study system, only two species (*Azteca* sp. 1 and *Crematogaster ampla*) fulfilled this criterion of dominance and never co‐occurred on the same individual tree. It is interesting to note that these two species are ecologically very similar, using similar nesting sites, occupying many nests per colony, foraging during both day and night, and have massive recruitment capacity and small individual body size. Moreover, these ant genera (*Azteca* and *Crematogaster*) are characterized by the presence of a modified proventriculus that enables them to harvest liquid food resources in a very effective way, reinforcing their dominance status (Davidson, 1997, 2005; Davidson, Cook, & Snelling, 2004).

The ant mosaic hypothesis is a specific predicated pattern of spatial structuring that emerges from competition over food in arboreal ant communities. Direct tests of this predicted pattern of competition‐driven spatial structuring have dominated the literature on community assembly in arboreal ant communities for many years (Blüthgen & Stork 2007; Jackson, 1984; Room, 1971). The central part of this hypothesis is that the whole habitat would be divided up into large mutually exclusive territories (Leston, 1973). We did not observe this pattern in our study. Moreover, one of the other expected patterns of the ant mosaic hypothesis is a well‐defined set of subdominant and subordinate ant species associated with each particular dominant ant species (Blüthgen et al. 2007; Majer, Delabie, & Smith, 1994; Room, 1971). This was also not seen in our analyses, with no detection of a particular set of species related to a given dominant ant species. Overall, our data showed support for a pattern of nonrandom spatial structuring, consistent with an important role of competition, but no evidence supporting the specific spatial structuring predicted by the ant mosaic hypotheses. These findings therefore advocate for future work to address the broader and more nuanced influences of competition on the spatial structuring of arboreal ant communities.

## 5 Conclusion

While our matrix‐level co‐occurrence analyses showed a random pattern of species associations with all ant species considered, we found a significant segregated pattern when using only the most common species. In addition, the pairwise analyses identified a significant number of nonrandom species associations among these most common ant species. Overall, our results suggest that these segregated associations are more consistent with competitive interactions between species driving species co‐occurrence patterns, with a limited influence of habitat selection. This result was further supported by our comparisons of important traits of the ant species that formed segregated pairs. Therefore, these findings provide support for an important role for trait‐mediated competitive interactions in determining coexistence in the focal arboreal ant community. Our analyses were unusual in the extent to which they were able to integrate a suite of potentially important environmental influences on assembly, providing greater certainty in interpreting the processes underlying the patterns we identified. We detected frequent segregation of the most common ant species. Moreover, despite the majority of the species pairs not forming significant associations and the lack of strong signs of habitat choices, we could find strong associations between biological characteristics and the significantly segregated or aggregated species pairs. Broadly, our findings suggest that trait‐mediated competitive interactions have more nuanced outcomes and spatial structuring in arboreal ant communities than has been previously considered. They also highlight the value of biodiversity inventory studies that explicitly incorporate potentially important environmental influences on assembly, and analyses that integrate such data.

## Conflict of Interest

None declared.

## References

[ece32606-bib-0001] Adler, P. B. , Salguero‐Gómez, R. , Compagnoni, A. , Hsu, J. S. , Ray‐Mukherjee, J. , Mbeau‐Ache, C. , & Franco, M. (2014). Functional traits explain variation in plant life history strategies. Proceedings of the National Academy of Sciences, 111, 740–745. 10.1073/pnas.1315179111 PMC3896207 24379395

[ece32606-bib-0002] Agrawal, A. A. , Ackerly, D. D. , Adler, F. , Arnold, A. E. , Cáceres, C. , Doak, D. F. , ··· Werner, E. (2007). Filling key gaps in population and community ecology. Frontiers in Ecology and the Environment, 5, 145–152.

[ece32606-bib-0003] Azeria, E. T. , Fortin, D. , Hébert, C. , Peres‐Neto, P. , Pothier, D. , & Ruel, J.‐C. (2009). Using null model analysis of species co‐occurrences to deconstruct biodiversity patterns and select indicator species. Diversity and Distributions, 15, 958–971.

[ece32606-bib-0004] Azeria, E. , Ibarzabal, J. , & Hébert, C. (2012). Effects of habitat characteristics and interspecific interactions on co‐occurrence patterns of saproxylic beetles breeding in tree boles after forest fire: Null model analyses. Oecologia, 168, 1123–1135. 22057900 10.1007/s00442-011-2180-0

[ece32606-bib-0005] Bestelmeyer, B. T. (2000). The trade‐off between thermal tolerance and behavioural dominance in a subtropical South American ant community. Journal of Animal Ecology, 69, 998–1009.

[ece32606-bib-0006] Blüthgen, N. , & Feldhaar, H. (2010). Food and shelter: How resources influence ant ecology In Lach L., Parr C. L. & Abbott K. L. (Eds.), Ant ecology, 1st ed. (pp. 115–136). Oxford: Oxford University Press.

[ece32606-bib-0007] Blüthgen, N. , Gebauer, G. , & Fiedler, K. (2003). Disentangling a rainforest food web using stable isotopes: Dietary diversity in a species‐rich ant community. Oecologia, 137, 426–435. 12898386 10.1007/s00442-003-1347-8

[ece32606-bib-0008] Blüthgen, N. , & Stork, N. E. (2007). Ant mosaics in a tropical rainforest in Australia and elsewhere: A critical review. Austral Ecology, 32, 93–104.

[ece32606-bib-0009] Cáceres, M. D. , & Legendre, P. (2009). Associations between species and groups of sites: Indices and statistical inference. Ecology, 90, 3566–3574. 20120823 10.1890/08-1823.1

[ece32606-bib-0010] Camarota, F. , Powell, S. , Vasconcelos, H. L. , Priest, G. , & Marquis, R. J. (2015). Extrafloral nectaries have a limited effect on the structure of arboreal ant communities in a Neotropical savanna. Ecology, 96, 231–240. 26236908 10.1890/14-0264.1

[ece32606-bib-0011] Campbell, H. , Fellowes, M. D. E. , & Cook, J. M. (2013). Arboreal thorn‐dwelling ants coexisting on the savannah ant‐plant, *Vachellia erioloba*, use domatia morphology to select nest sites. Insectes sociaux, 60, 373–382.

[ece32606-bib-0012] Campbell, H. , Fellowes, M. D. , & Cook, J. M. (2015). The curious case of the camelthorn: Competition, coexistence, and nest‐site limitation in a multispecies mutualism. The American Naturalist, 186, E172–E181. 10.1086/683462 26655993

[ece32606-bib-0013] Campos, R. I. , Vasconcelos, H. L. , Ribeiro, S. P. , Neves, F. S. , & Soares, J. P. (2006). Relationship between tree size and insect assemblages associated with *Anadenanthera macrocarpa* . Ecography, 29, 442–450.

[ece32606-bib-0014] Cerdá, X. , Arnan, X. , & Retana, J. (2013). Is competition a significant hallmark of ant (Hymenoptera: Formicidae) ecology? Myrmecological News, 18, 131–147.

[ece32606-bib-0015] Cerdá, X. , Retana, J. , & Cros, S. (1997). Thermal disruption of transitive hierarchies in mediterranean ant communities. Journal of Animal Ecology, 66, 363–374.

[ece32606-bib-0016] Cerdá, X. , Retana, J. , & Manzaneda, A. (1998). The role of competition by dominants and temperature in the foraging of subordinate species in mediterranean ant communities. Oecologia, 117, 404–412. 10.1007/s004420050674 28307920

[ece32606-bib-0017] Chase, J. M. , & Leibold, M. A. (2003). Ecological niches. Chicago, IL: University of Chicago Press.

[ece32606-bib-0018] Chesson, P. (2000). Mechanisms of maintenance of species diversity. Annual Review of Ecology and Systematics, 31, 343–358.

[ece32606-bib-0019] Connor, E. F. , & Simberloff, D. (1979). The assembly of species communities: Chance or competition? Ecology, 60, 1132–1140.

[ece32606-bib-0020] Connor, E. F. , & Simberloff, D. (1984). Neutral models of species’ co‐occurrence patterns In Strong D. R., Simberloff D., Abele L. G. & Thistle A. B. (Eds.), Ecological communities: Conceptual issues and the evidence (pp. 316–331). Princeton, NJ: Princeton University Press.

[ece32606-bib-0021] Davidson, D. W. (1997). The role of resource imbalances in the evolutionary ecology of tropical arboreal ants. Biological Journal of the Linnean Society, 61, 153–181.

[ece32606-bib-0022] Davidson, D. (2005). Ecological stoichiometry of ants in a New World rain forest. Oecologia, 142, 221–231. 15503164 10.1007/s00442-004-1722-0

[ece32606-bib-0023] Davidson, D. , Cook, S. , & Snelling, R. (2004). Liquid‐feeding performances of ants (Formicidae): Ecological and evolutionary implications. Oecologia, 139, 255–266. 15034777 10.1007/s00442-004-1508-4

[ece32606-bib-0024] Davidson, D. W. , Cook, S. C. , Snelling, R. R. , & Chua, T. H. (2003). Explaining the abundance of ants in lowland tropical rainforest canopies. Science, 300, 969–972. 12738862 10.1126/science.1082074

[ece32606-bib-0025] Dejean, A. , Fisher, B. L. , Corbara, B. , Rarevohitra, R. , Randrianaivo, R. , Rajemison, B. , & Leponce, M. (2010). Spatial distribution of dominant arboreal ants in a malagasy coastal rainforest: Gaps and presence of an invasive species. PLoS One, 5, e9319. 20174474 10.1371/journal.pone.0009319 PMC2824834

[ece32606-bib-0026] Diamond, J. (1975). Assembly of species communities In Cody M. L. & Diamond J. M. (Eds.), Ecology and evolution of communities (pp. 342–344). Cambridge, MA: Harvard University Press.

[ece32606-bib-0027] Diamond, J. , & Gilpin, M. (1982). Examination of the “null” model of Connor and Simberloff for species co‐occurrences on Islands. Oecologia, 52, 64–74. 10.1007/BF00349013 28310110

[ece32606-bib-0028] Dice, L. R. (1945). Measures of the amount of ecologic association between species. Ecology, 26, 297–302.

[ece32606-bib-0029] Djiéto‐Lordon, C. , Dejean, A. , Gibernau, M. , Hossaert‐McKey, M. , & McKey, D. (2004). Symbiotic mutualism with a community of opportunistic ants: Protection, competition, and ant occupancy of the myrmecophyte *Barteria nigritana* (Passifloraceae). Acta Oecologica, 26, 109–116.

[ece32606-bib-0030] Dufrene, M. , & Legendre, P. (1997). Species assemblages and indicator species: The need for a flexible asymmetrical approach. Ecological Monographs, 67, 345–366.

[ece32606-bib-0031] Fellers, J. H. (1987). Interference and exploitation in a guild of woodland ants. Ecology, 68, 1466–1478.

[ece32606-bib-0032] Fowler, D. , Lessard, J.‐P. , & Sanders, N. J. (2014). Niche filtering rather than partitioning shapes the structure of temperate forest ant communities. Journal of Animal Ecology, 83, 943–952. 24289457 10.1111/1365-2656.12188

[ece32606-bib-0033] Gotelli, N. J. (2000). Null model analysis of species co‐occurrence patterns. Ecology, 81, 2606–2621.

[ece32606-bib-0034] Gotelli, N. J. (2001). Research frontiers in null model analysis. Global Ecology & Biogeography, 10, 337–343.

[ece32606-bib-0035] Gotelli, N. J. , & Ellison, A. M. (2002). Assembly rules for New England ant assemblages. Oikos, 99, 591–599.

[ece32606-bib-0036] Gotelli, N. J. , & Entsminger, G. (2001). Swap and fill algorithms in null model analysis: Rethinking the knight's tour. Oecologia, 129, 281–291. 10.1007/s004420100717 28547607

[ece32606-bib-0037] Gotelli, N. J. , & Entsminger, G. L. (2003). Swap algorithms in null model analysis. Ecology, 84, 532–535. 10.1007/s004420100717 28547607

[ece32606-bib-0038] Gotelli, N. J. , & Graves, G. R. (1996). Null models in ecology: Linking classic and contemporary approaches. Washington, DC: Smithsonian Institution Press.

[ece32606-bib-0039] Gotelli, N. J. , & McCabe, D. J. (2002). Species co‐occurrence: A meta‐analysis of j. m. diamond's assembly rules model. Ecology, 83, 2091–2096.

[ece32606-bib-0040] Gotelli, N. J. , & Ulrich, W. (2012). Statistical challenges in null model analysis. Oikos, 121, 171–180.

[ece32606-bib-0041] Hölldobler, B. , & Wilson, E. (1990). The ants. Cambridge, MA: Harvard University Press.

[ece32606-bib-0042] Hutchinson, G. E. (1959). Homage to Santa‐Rosalia or why are there so many kinds of animals. American Naturalist, 93, 145–159.

[ece32606-bib-0043] Jackson, D. A. (1984). Ant distribution patterns in a cameroonian cocoa plantation: Investigation of the ant mosaic hypothesis. Oecologia, 62, 318–324. 10.1007/BF00384263 28310884

[ece32606-bib-0044] Klimes, P. , Idigel, C. , Rimandai, M. , Fayle, T. M. , Janda, M. , Weiblen, G. D. , & Novotny, V. (2012). Why are there more arboreal ant species in primary than in secondary tropical forests? Journal of Animal Ecology, 81, 1103–1112. 22642689 10.1111/j.1365-2656.2012.02002.x

[ece32606-bib-0045] Koch, E. B. A. , Camarota, F. , & Vasconcelos, H. L. (2016). Plant ontogeny as a conditionality factor in the protective effect of ants on a neotropical tree. Biotropica, 48, 198–205.

[ece32606-bib-0046] Lach, L. , Parr, C. L. , & Abbot, K. L. (2010). Ant ecology. Oxford: Oxford University Press.

[ece32606-bib-0047] Lessard, J. P. , Dunn, R. , & Sanders, N. (2009). Temperature‐mediated coexistence in temperate forest ant communities. Insectes Sociaux, 56, 149–156.

[ece32606-bib-0048] Leston, D. (1970). Entomology of cocoa farm. Annual Review of Entomology, 15, 273–294.

[ece32606-bib-0049] Leston, D. (1973). The ant mosaic‐tropical tree crops and the limiting of pests and diseases. PANS Pest Articles & News Summaries, 19, 311–341.

[ece32606-bib-0050] Levine, J. M. , & HilleRisLambers, J. (2009). The importance of niches for the maintenance of species diversity. Nature, 461, 254–257. 19675568 10.1038/nature08251

[ece32606-bib-0051] MacArthur, R. , & Levins, R. (1967). The limiting similarity, convergence, and divergence of coexisting species. The American Naturalist, 101, 377–385.

[ece32606-bib-0052] Majer, J. D. (1972). The ant mosaic in Ghana cocoa farms. Bulletin of Entomological Research, 62, 151–160.

[ece32606-bib-0053] Majer, J. D. , Delabie, J. H. C. , & Smith, M. R. B. (1994). Arboreal ant community patterns in Brazilian cocoa farms. Biotropica, 26, 73–83.

[ece32606-bib-0054] Moreno, M. I. C. (2005). Estado nutricional de espécies lenhosas e disponibilidade de nutrientes no solo e na serapilheira em diferentes fitofisionomias do cerrado na região do triângulo mineiro, Master Thesis. Universidade de Brasília, Brasília.

[ece32606-bib-0055] Myers, N. , Mittermeier, R. A. , Mittermeier, C. G. , Da Fonseca, G. A. , & Kent, J. (2000). Biodiversity hotspots for conservation priorities. Nature, 403, 853–858. 10706275 10.1038/35002501

[ece32606-bib-0056] Oksanen, J. , Kindt, R. , Legendre, P. , O'Hara, B. , & Stevens, M. H. H. (2013). VEGAN: Community ecology package (R package version 3.3‐3). Vienna, Austria: R Foundation for Statistical Computing.

[ece32606-bib-0057] Oliveira‐Filho, A. T. , & Ratter, J. A. (2002). Vegetation physiognomies and woody flora of the cerrado biome. In P. S. Oliveira & R. J. Marquis (Eds.), The Cerrados of Brazil: Ecology and Natural History of a Neotropical Savanna (pp.91–120). New York: Columbia University Press.

[ece32606-bib-0058] Pacheco, R. , & Vasconcelos, H. L. (2012). Habitat diversity enhances ant diversity in a naturally heterogeneous Brazilian landscape. Biodiversity and Conservation, 21, 797–809.

[ece32606-bib-0059] Parr, C. L. , & Gibb, H. (2010). Competition and the role of dominant ants In Lach L., Parr C. L. & Abbot K. L. (Eds.), Ant ecology (pp. 77–96). Oxford: Oxford University Press.

[ece32606-bib-0060] Parr, C. L. , & Gibb, H. (2012). The discovery–dominance trade‐off is the exception, rather than the rule. Journal of Animal Ecology, 81, 233–241. 21854375 10.1111/j.1365-2656.2011.01899.x

[ece32606-bib-0061] Peres‐Neto, P. R. , Olden, J. D. , & Jackson, D. A. (2001). Environmentally constrained null models: Site suitability as occupancy criterion. Oikos, 93, 110–120.

[ece32606-bib-0062] Pfeiffer, M. , Cheng Tuck, H. , & Chong Lay, T. (2008). Exploring arboreal ant community composition and co‐occurrence patterns in plantations of oil palm *Elaeis guineensis* in Borneo and Peninsular Malaysia. Ecography, 31, 21–32.

[ece32606-bib-0063] Philpott, S. M. , & Foster, P. F. (2005). Nest‐site limitation in coffee agroecosystems: Artificial nests maintain diversity of arboreal ants. Ecological Applications, 15, 1478–1485.

[ece32606-bib-0064] Pitta, E. , Giokas, S. , & Sfenthourakis, S. (2012). Significant pairwise co‐occurrence patterns are not the rule in the majority of biotic communities. Diversity, 4, 179–193.

[ece32606-bib-0065] Powell, S. , Costa, A. N. , Lopes, C. T. , & Vasconcelos, H. L. (2011). Canopy connectivity and the availability of diverse nesting resources affect species coexistence in arboreal ants. Journal of Animal Ecology, 80, 352–360. 21118199 10.1111/j.1365-2656.2010.01779.x

[ece32606-bib-0066] R Development Core Team (2014). R: A language and environment for statistical computing. R foundation for statistical computing Retrieved from http://www.r-project.org

[ece32606-bib-0067] Ribas, C. R. , & Schoereder, J. H. (2002). Are all ant mosaics caused by competition?. Oecologia, 131, 606–611. 10.1007/s00442-002-0912-x 28547556

[ece32606-bib-0068] Ribas, C. R. , Schoereder, J. H. , Pic, M. , & Soares, S. M. (2003). Tree heterogeneity, resource availability, and larger scale processes regulating arboreal ant species richness. Austral Ecology, 28, 305–314.

[ece32606-bib-0069] Room, P. M. (1971). The relative distributions of ant species in ghana's cocoa farms. Journal of Animal Ecology, 40, 735–751.

[ece32606-bib-0070] Russell, J. A. , Moreau, C. S. , Goldman‐Huertas, B. , Fujiwara, M. , Lohman, D. J. , & Pierce, N. E. (2009). Bacterial gut symbionts are tightly linked with the evolution of herbivory in ants. Proceedings of the National Academy of Sciences, 106, 21236–21241. 10.1073/pnas.0907926106 PMC2785723 19948964

[ece32606-bib-0071] Sanders, N. J. , Crutsinger, G. M. , Dunn, R. R. , Majer, J. D. , & Delabie, J. H. C. (2007). An ant mosaic revisited: Dominant ant species disassemble arboreal ant communities but co‐occur randomly. Biotropica, 39, 422–427.

[ece32606-bib-0072] Sanders, N. J. , Gotelli, N. J. , Wittman, S. E. , Ratchford, J. S. , Ellison, A. M. , & Jules, E. S. (2007). Assembly rules of ground‐foraging ant assemblages are contingent on disturbance, habitat and spatial scale. Journal of Biogeography, 34, 1632–1641.

[ece32606-bib-0073] Sanderson, J. G. (2004). Null model analysis of communities on gradients. Journal of Biogeography, 31, 879–883.

[ece32606-bib-0074] Schoener, T. W. (1974). Resource partitioning in ecological communities. Science, 185, 27–39. 17779277 10.1126/science.185.4145.27

[ece32606-bib-0075] Sfenthourakis, S. , Tzanatos, E. , & Giokas, S. (2006). Species co‐occurrence: The case of congeneric species and a causal approach to patterns of species association. Global Ecology and Biogeography, 15, 39–49.

[ece32606-bib-0076] Stone, L. , & Robert, A. (1990). The checkerboard score and species distributions. Oecologia, 85, 74–79. 10.1007/BF00317345 28310957

[ece32606-bib-0077] Stringer, L. D. , Haywood, J. , & Lester, P. J. (2007). The influence of temperature and fine‐scale resource distribution on resource sharing and domination in an ant community. Ecological Entomology, 32, 732–740.

[ece32606-bib-0078] Strong, D. R. , Simberloff, D. , Abele, L. G. , & Thistle, A. B. (1984). Ecological communities: Conceptual issues and the evidence. Princeton, NJ: Princeton University Press.

[ece32606-bib-0079] Stuble, K. L. , Rodriguez‐Cabal, M. A. , McCormick, G. L. , Jurić, I. , Dunn, R. R. , & Sanders, N. J. (2013). Tradeoffs, competition, and coexistence in eastern deciduous forest ant communities. Oecologia, 171, 981–992. 23242423 10.1007/s00442-012-2459-9

[ece32606-bib-0080] Sutherland, W. J. , Freckleton, R. P. , Godfray, H. C. J. , Beissinger, S. R. , Benton, T. , Cameron, D. D. , ··· Wiegand, T. (2013). Identification of 100 fundamental ecological questions. Journal of Ecology, 101, 58–67.

[ece32606-bib-0081] Ulrich, W. , & Gotelli, N. J. (2013). Pattern detection in null model analysis. Oikos, 122, 2–18.

[ece32606-bib-0082] Veech, J. A. (2014). The pairwise approach to analysing species co‐occurrence. Journal of Biogeography, 41, 1029–1035.

[ece32606-bib-0083] Wittman, S. E. , Sanders, N. J. , Ellison, A. M. , Jules, E. S. , Ratchford, J. S. , & Gotelli, N. J. (2010). Species interactions and thermal constraints on ant community structure. Oikos, 119, 551–559.

