# Supplementary material for: Co‐occurrence patterns in a diverse arboreal ant community are explained more by competition than habitat requirements
Source: Ecol Evol. 2016 Nov 23;6(24):8907–18. doi: 10.1002/ece3.2606 (PMC5192950; doi:10.1002/ece3.2606)
Supplement: Supplementary file 1 [file ECE3-6-8907-s001.docx]

**Supporting Information**

**Table S 1:** List of ant species/ morphospecies, divided by the genera and the subfamily they belong to, together with number of trees were these ant species were collected in this study. (Species with an * are those amongst the 14 most common).

|  |  |  |
| --- | --- | --- |
| Ant species |  | Number of trees |
|  |  |  |
| **DOLICHODERINAE** |  |  |
|  |  |  |
| *Azteca* sp. 1* |  | 55 |
| *Azteca* sp. 3 |  | 2 |
| *Dolichoderus bispinosus* (Olivier, 1792) |  | 4 |
| *Dolichoderus lamellosus* (Mayr, 1870) |  | 12 |
| *Dolichoderus lutosus* (Smith, 1858) |  | 15 |
| *Dorymyrmex* sp. 1 |  | 5 |
| *Dorymyrmex* sp. 2 |  | 2 |
| *Tapinoma* sp. 1* |  | 62 |
| **ECTATOMMINAE** |  |  |
|  |  |  |
| *Ectatomma tuberculatum* (Olivier, 1792) |  | 1 |
| *Gnamptogenys* sp. 1 |  | 1 |
| **FORMICINAE** |  |  |
|  |  |  |
| *Brachymyrmex* sp. 1 |  | 3 |
| *Camponotus arboreus* (Smith, 1858) |  | 6 |
| *Camponotus atriceps** (Smith, 1858) |  | 81 |
| *Camponotus balzani* Emery, 1894 |  | 3 |
| *Camponotus blandus* (Smith, 1858) |  | 9 |
| *Camponotus bonariensis** Mayr, 1868 |  | 123 |
| *Camponotus cingulatus* Mayr, 1862 |  | 14 |
| *Camponotus fastigatus* Roger, 1863 |  | 3 |
| *Camponotus innocens* Forel, 1909 |  | 1 |
| *Camponotus lespesii* Forel, 1886 |  | 3 |
| *Camponotus melanoticus** Emery, 1894 |  | 28 |
| *Camponotus senex** (Smith, 1858) |  | 164 |
| *Camponotus sericeiventris** Wheeler, 1931 |  | 34 |
| *Camponotus* sp. 15 |  | 13 |
| *Camponotus* sp. 20 |  | 2 |
| *Camponotus* sp. 37 |  | 10 |
| *Camponotus* sp. 58 |  | 11 |
| *Camponotus* sp. 6 |  | 14 |
| *Camponotus* sp. 65 |  | 2 |
| *Myrmelachista* sp. 1 |  | 4 |
| *Myrmelachista* sp. 2 |  | 1 |
| **MYRMICINAE** |  |  |
|  |  |  |
| *Atta laevigata* (Smith, 1858) |  | 3 |
| *Cephalotes adolphi* (Emery, 1906) |  | 1 |
| *Cephalotes angustus* (Mayr, 1862) |  | 2 |
| *Cephalotes atratus* (Linnaeus, 1758) |  | 3 |
| *Cephalotes clypeatus* (Fabricius, 1804) |  | 1 |
| *Cephalotes cordatus* (Santschi, 1921) |  | 2 |
| *Cephalotes depressus* (Klug, 1824) |  | 1 |
| *Cephalotes eduarduli* (Forel, 1921) |  | 1 |
| *Cephalotes grandinosus* (Smith, 1860) |  | 9 |
| *Cephalotes liepini* De Andrade & Baroni Urbani 1999 |  | 2 |
| *Cephalotes maculatus* (Smith, 1876) |  | 4 |
| *Cephalotes minutus* (Fabricius, 1804) |  | 2 |
| *Cephalotes pallidoides* De Andrade & Baroni Urbani 1999 |  | 4 |
| *Cephalotes pellans* De Andrade & Baroni Urbani 1999 |  | 5 |
| *Cephalotes persimilis* De Andrade & Baroni Urbani 1999 |  | 7 |
| *Cephalotes pusillus** Klug 1824 |  | 131 |
| *Cephalotes specullaris* Brandão, Feitosa, Powell & Del-Claro 2014 | | 2 |
| *Crematogaster ampla** Forel, 1912 |  | 20 |
| *Crematogaster* sp. 2 |  | 2 |
| *Crematogaster* sp. 4 |  | 1 |
| *Nesomyrmex asper* (Mayr, 1887) |  | 1 |
| *Nesomyrmex* sp. 2 |  | 3 |
| *Pheidole* sp. 1 |  | 5 |
| *Pheidole* sp. 3 |  | 5 |
| *Pheidole* sp. 6 |  | 5 |
| *Pheidole* sp. 7 |  | 2 |
| *Solenopsis* sp. 1* |  | 21 |
| *Solenopsis* sp. 2 |  | 3 |
| *Wasmmania* *lutzi* Forel, 1908 |  | 7 |
| **PONERINAE** |  |  |
|  |  |  |
| *Hypoponera* sp. 1 |  | 1 |
| *Neoponera villosa* (Fabricius, 1804) |  | 13 |
| **PSEUDOMYRMICINAE** |  |  |
|  |  |  |
| *Pseudomyrmex curacaensis** (Forel, 1912) |  | 44 |
| *Pseudomyrmex elongatus** (Mayr, 1870) |  | 28 |
| *Pseudomyrmex gracilis** (Fabricius, 1804) |  | 149 |
| *Pseudomyrmex kuenckeli* (Emery, 1890) |  | 4 |
| *Pseudomyrmex maculatus* (Smith, 1855) |  | 2 |
| *Pseudomyrmex lizeri* (Sanstchi, 1922) |  | 2 |
| *Pseudomyrmex sericeus* (Mayr, 1870) |  | 3 |
| *Pseudomyrmex simplex* (Smith, 1877) |  | 7 |
| *Pseudomyrmex* sp. 10 |  | 2 |
| *Pseudomyrmex* sp. 11 |  | 2 |
| *Pseudomyrmex tenuissimus* (Emery, 1906) |  | 10 |
| *Pseudomyrmex unicolor* (Smith, 1855) |  | 3 |
| *Pseudomyrmex urbanus** (Smith, 1877) |  | 20 |
|  |  |  |
|  |  |  |

**Table S 2:** Pairwise co-occurrence patterns of the 14 most common ant species in our study. S= segregated associations, A= aggregated associations, R= random associations. Empty spaces mean that the significance status of the relationship presented in the unconstrained null model did not change under the habitat constrained models.

|  |  |  |  |  |  |  |  |  |
| --- | --- | --- | --- | --- | --- | --- | --- | --- |
| Species pairs | Unconstrained | | Habitat constrained | | | | | |
|  |  |  |  |  |  |  |  |  |
|  |  |  | plant species | | plant size | | vegetation structure | |
|  |  |  |  |  |  |  |  |  |
|  |  |  |  |  |  |  |  |  |
|  |  |  |  |  |  |  |  |  |
| *Ca. senex*X *Ce. pusillus* |  | S |  |  |  |  |  |  |
| *Ce. pusillus*X *Cr. ampla* |  | S |  |  |  |  |  |  |
| *Ca. bonariensis*X *Ca. atriceps* |  | S |  |  |  |  |  |  |
| *Ca. bonariensis*X *Ca. sericeiventris* |  | S |  |  |  |  |  |  |
| *Azteca* sp. 1 X *Solenopsis* sp. 1 |  | S |  |  |  |  |  |  |
| *Tapinoma* sp. 1 X *Azteca* sp. 1 |  | S |  |  |  |  |  |  |
| *Azteca* sp. 1 X *Cr. ampla* |  | S |  |  |  |  |  |  |
| *P. gracilis*X *P. urbanus* |  | S |  |  |  |  |  |  |
| *Ce. pusillus*X *Azteca* sp. 1 |  | S |  |  |  |  |  |  |
| *Ca. atriceps*X *Ca. melanoticus* |  | S |  |  |  |  |  |  |
| *P. curacauensis*X *P. urbanus* |  | S |  |  |  |  |  |  |
| *P. gracilis*X *Azteca* sp. 1 |  | S |  | R |  | R |  | R |
| *Ca. atriceps*X *Tapinoma* sp. 1 |  | R |  |  |  |  |  |  |
| *Ce. pusillusX Ca. atriceps* |  | R |  |  |  |  |  |  |
| *P. gracilis*X *Tapinoma* sp. 1 |  | R |  | A |  | A |  |  |
| *Tapinoma* sp. 1 X *Ca. sericeiventris* |  | A |  |  |  |  |  |  |
| *Ca. bonariensis*X *P. urbanus* |  | A |  |  |  |  |  |  |
| *Ca. atriceps*X *Ca. sericeiventris* |  | A |  |  |  |  |  |  |
| *Ca. senex*X *Ca. sericeiventris* |  | A |  | R |  |  |  |  |
| *P. gracilis*X *Ce. pusillus* |  | A |  |  |  |  |  |  |
| *Ce. pusillus*X *Ca. bonariensis* |  | A |  | R |  |  |  |  |
| *Ce. pusillus*X *P. curacaensis* |  | A |  |  |  |  |  |  |
|  |  |  |  |  |  |  |  |  |
|  |  |  |  |  |  |  |  |  |

**Table S3:** Species characteristics in common inside each significant species pair on a) segregated pairs and b) aggregated pairs:

|  |  |  |  |  |  |
| --- | --- | --- | --- | --- | --- |
| Species pairs | Species biological characteristics | | | | |
|  |  |  |  |  |  |
|  | Nesting ecology | |  | Other | |
|  |  |  |  |  |  |
|  | Both have same nest structure? | Both have extensive cavity use? |  | Both have same activity period? | Both have same recruiting strategy? |
|  |  |  |  |  |  |
|  |  |  |  |  |  |
| a) Segregated pairs |  |  |  |  |  |
|  |  |  |  |  |  |
| *Azteca* sp. 1 X *Ce. pusillus* | yes | yes |  | yes | no |
| *Azteca* sp. 1 X *Cr. ampla* | yes | yes |  | yes | yes |
| *Azteca* sp. 1 X *Solenopsis* sp. 1 | no | yes |  | yes | yes |
| *Azteca* sp. 1 X *Tapinoma* sp. 1 | no | no |  | yes | yes |
| *Ca. atriceps* X *Ca. melanoticus* | no | no |  | yes | no |
| *Ca. bonariensis* X *Ca. atriceps* | no | no |  | yes | yes |
| *Ca. bonariensis* X *Ca. sericeiventris* | no | no |  | no | no |
| *Ca. senex* X *Ce. pusillus* | no | no |  | no | no |
| *Ce. pusillus* X *Cr. ampla* | yes | yes |  | yes | no |
| *P. curacauensis* X *P. urbanus* | yes | no |  | yes | yes |
| *P. gracilis* X *P. urbanus* | yes | no |  | yes | yes |
|  |  |  |  |  |  |
| b) Aggregated pairs |  |  |  |  |  |
|  |  |  |  |  |  |
| *Ca. atriceps* X *Ca. sericeiventris* | no | no |  | no | no |
| *Ca. bonariensis* X *P. urbanus* | no | no |  | no | no |
| *Ce. pusillus* X *P. curacaensis* | no | no |  | no | no |
| *P. gracilis* X *Ce. pusillus* | no | no |  | no | no |
| *Tapinoma* sp. 1 X *Ca. sericeiventris* | no | no |  | yes | no |
|  |  |  |  |  |  |
|  |  |  |  |  |  |
